# Serum level of calpains product as a novel biomarker of acute lung injury following cardiopulmonary bypass

**DOI:** 10.3389/fcvm.2022.1000761

**Published:** 2022-11-16

**Authors:** Chenlong Yi, Fangyu Chen, Rongrong Ma, Zhi Fu, Meijuan Song, Zhuan Zhang, Lingdi Chen, Xing Tang, Peng Lu, Ben Li, Qingfen Zhang, Qifeng Song, Guangzheng Zhu, Wei Wang, Qiang Wang, Xiaowei Wang

**Affiliations:** ^1^Department of Cardiovascular Surgery, The Affiliated Hospital of Yangzhou University, Yangzhou University, Yangzhou, China; ^2^Department of Thoracic and Cardiovascular Surgery, The First Affiliated Hospital of Nanjing Medical University, Nanjing, China; ^3^Department of Thoracic and Cardiovascular Surgery, Dalian Medical University, Dalian, China; ^4^Department of Anesthesiology, The Affiliated Hospital of Yangzhou University, Yangzhou University, Yangzhou, China; ^5^Jiangsu Provincial Key Laboratory of Geriatrics, Department of Geriatrics, The First Affiliated Hospital, Nanjing Medical University, Nanjing, China; ^6^Department of Operating Theatre, The Affiliated Hospital of Yangzhou University, Yangzhou University, Yangzhou, China; ^7^Department of Anesthesiology, Peking University People’s Hospital, Beijing, China

**Keywords:** acute lung injury, calpains, cardiopulmonary bypass (CPB), PaO_2_/FiO_2_, heart surgery

## Abstract

**Objective:**

The aim of this study was to test the hypothesis whether serum level of calpains could become a meaningful biomarker for diagnosis of acute lung injury (ALI) in clinical after cardiac surgery using cardiopulmonary bypass (CPB) technology.

**Methods and results:**

Seventy consecutive adults underwent cardiac surgery with CPB were included in this prospective study. Based on the American-European Consensus Criteria (AECC), these patients were divided into ALI (*n* = 20, 28.57%) and non-ALI (*n* = 50, 71.43%) groups. Serum level of calpains in terms of calpains’ activity which was expressed as relative fluorescence unit (RFU) per microliter and measured at beginning of CPB (baseline), 1 h during CPB, end of CPB as well as 1, 12, and 24 h after CPB. Difference of serum level of calpains between two groups first appeared at the end of CPB and remained different at subsequent test points. Univariate and multivariate logistic regression analysis indicated that serum level of calpains 1 h after CPB was an independent predictor for postoperative ALI (OR 1.011, 95% CI 1.001, 1.021, *p* = 0.033) and correlated with a lower PaO_2_/FiO_2_ ratio in the first 2 days (The first day: r = -0.389, *p* < 0.001 and the second day: r = -0.320, *p* = 0.007) as well as longer mechanical ventilation time (r = 0.440, *p* < 0.001), intensive care unit (ICU) length of stay (LOS) (r = 0.419, *p* < 0.001) and hospital LOS (r = 0.297, *p* = 0.013).

**Conclusion:**

Elevated serum level of calpains correlate with impaired lung function and poor clinical outcomes, indicating serum level of calpains could act as a potential biomarker for postoperative ALI following CPB in adults.

**Clinical trial registration:**

[https://clinicaltrials.gov/show/NCT05610475], identifier [NCT05610475].

## Introduction

More than two million patients worldwide receive heart surgery every year, majority of these surgical patients will undergo cardiopulmonary bypass (CPB) (1). With the improvement of surgical technology and CPB technology, the incidence of complications after CPB is getting lower. However, the incidence of postoperative acute lung injury (ALI) due to CPB is still as high as 20–35% compared with non-CPB surgery (2, 3). Postoperative lung injury usually leads to the extension of Intensive Care Unit (ICU) and total hospital stay, thereby increasing the mental and economic burden of patients. If severe cases evolve into acute respiratory distress syndrome (ARDS), the mortality rate is close to 30% (4, 5). According to clinical experience, the earlier lung damage is detected, the more successful the treatment will be (6–8). Therefore, in-depth study of the mechanism of CPB-related lung injury, discovery, and validation of certain alarming biomarker of CPB-related lung injury in cardiac surgery patients will greatly contribute to early diagnosis and effective treatment decisions making.

The pathophysiological changes of lung injury caused by CPB are mainly manifested in the inflammatory reaction and ischemia-reperfusion injury (9–12). Systemic inflammation causes the aggregation and activation of neutrophils in small pulmonary vessels. At the same time, vascular permeability increases, and pulmonary interstitial edema occurs, which affects oxygen diffusion, leads to lung dysfunction (11, 13). Two sets of blood supply systems exist in the lungs, including the pulmonary artery system and the bronchial artery system. The former is the main blood supply to the lungs which accounts for 97% of the pulmonary blood flow, while bronchial artery system accounts for only 3% of pulmonary blood flow. Complete cessation of the pulmonary circulatory system during CPB results in a significant decrease in pulmonary blood flow, a feature that makes lung tissue more vulnerable to ischemia-reperfusion injury (14). It is worth mentioning that both pathological processes can activate calpains (15, 16).

Calpain family is a large protease family composed of at least 15 different subtypes, some members are expressed widely in organisms, known as traditional calpains, while other members show tissue specificity (17). According to the level of Ca^2+^ activation concentration, traditional calpains are divided into calpain-1 (micromolar level) and calpain-2 (millimolar level), which are the most characteristic members of calpain family and ubiquitously expressed (17). Under physiological conditions, the macromolecules of calpains prevent them from freely passing through the cell membrane. Therefore, calpains play a biological role primarily in the cytoplasm (18). However, more and more studies reported that actived calpains were found extracellularly and affected cellular activities (19). The purpose of this prospective study was to examine changes in serum level of traditional calpains in patients following CPB and to explore whether serum calpains could serve as a potential biological marker for predicting the incidence of postoperative ALI.

## Methods

### Study population

This prospective study was performed in Affiliated Hospital of Yangzhou University, China. A total of 70 patients beyond the age of 18 who underwent cardiac surgery using CPB technology at cardiovascular surgery department between February 2022 and July 2022 were enrolled in this trial (Supplementary Figure 1). The study protocols were approved by the Ethics Committee of Affiliated Hospital of Yangzhou University (2022-YKL03-SKJ013). Exclusion criteria: Presence of abnormal liver, kidney or other organ function, pulmonary inflammation, COPD or tumors, postoperative need for extracorporeal membrane oxygenation support, or patients who refused to participate were excluded.

### Data collection

Demographic data contains patient’s gender, age, body mass index (BMI), smoking, hypertension, diabetes mellitus, cardiac function and left ventricular ejection fraction (LVEF) were recorded. Routine hemodynamics were performed in all patients during the surgical procedure. Arterial blood gas surveillance was performed every 30 min after mechanical ventilation until the end of the operation. The ratio of inspiratory oxygen fraction to oxygen pressure (PaO_2_/FiO_2_) was calculated. Intraoperative data such as surgery type, operation time, CPB time, clamp time, and ultrafiltered volume removal (UVR), as well as serum samples at different time points were collected. After operation, the patients were transferred to the ICU immediately and given sedation, intubation, and mechanical ventilation. Laboratory parameters of all postoperative patients including blood cell analysis, blood gas surveillance, liver, and kidney function alone with chest radiograph and echocardiography were dynamically monitored. A structured tutorial was used to establish consensus in the interpretation of radiographs for radiographic lung-injury scores (LISs) (20, 21). In addition, mechanical ventilation (MV) time together with length of ICU stay and hospital stay were followed up.

### Main definitions

Based on the American-European Consensus Conference (AECC) definition (22). Enrolled patients (*n* = 70) were divided into the ALI group (*n* = 20) and non-ALI group (*n* = 50). ALI was defined as follows: Oxygenation: PaO_2_/FiO_2_ < 300 mmHg (ignoring positive end-expiratory pressure); Chest radiograph: Visible bilateral infiltration; Pulmonary artery occlusion pressure (PAOP): Below 18 mmHg and no cardiogenic pulmonary edema (CPE). (CPE can be determined by PAOP over 18 mmHg or by the presence of central venous pressure over 14 mmHg with LVEF below 45%).

### Determination of serum level of calpains

Serial serum samples were obtained at the following time points: beginning of CPB (baseline), 1 h during CPB, the end of CPB as well as 1, 12, and 24 h after CPB. After centrifugation at 3,000 rpm for 15 min at 4^°^C, serum was aliquoted and frozen at −80^°^C until assayed. Serum level of calpains reacted with calpains’ activity, which was measured by using a fluorometric kit (Abcam, Cambridge, UK) according to the manufacturer’s protocol. Calpains’ activity was analyzed using a fluorometer equipped with a 400 nm excitation filter and a 505 nm emission filter. Laboratory staff were blinded to clinical parameters, and researchers involved in collecting demographic and preoperative data were blinded to the level of calpains. The activity of calpains was expressed as relative fluorescence unit (RFU) per microliter of serum per sample.

### Statistical analysis

The data were expressed as mean (standard deviation) or median (25th percentile, 75th percentile). All statistical analyses in this study were performed with SPSS 26.0 for Windows (SPSS Inc., Chicago, IL, USA). After normal distribution analysis by one sample Kolmogorov–Smirnov test. Unpaired student’s t test was devoted to further analyze the normally distributed data while Mann–Whitney U test was used to further analyze the abnormally distributed data. χ^2^ test was employed to compare categorical data as appropriate. Pearson or Spearman correlation test were performed to access the correlation between serum level of calpains and clinical parameters such as CPB time, PaO_2_/FiO_2_ ratio, MV time, ICU LOS, and hospital LOS. Receiver operating characteristic curve (ROC) was computed, and area under the curve (AUC) was calculated to evaluate the significance of calpains’ level in diagnosing ALI. Risk factors associated to the development of ALI including cardiac function, operation time, CPB time, clamp time, serum level of calpains at the end of CPB and serum level of calpains at 1 h after CPB were introduced into further univariate analysis. Multivariate logistic regression analysis (Enter) was then performed on statistically significant univariate predictors to determine the independent risk factor for postoperative ALI. Statistical significance was identified as *p* < 0.05.

## Results

### Demographic characteristics

A total of 70 patients met the inclusion criteria. Among them, postoperative ALI occurred in 20 cases (28.57%). The demographic and perioperative characteristics of all participants are shown in Table 1. Demographic parameters of gender, age, BMI, smoking, hypertension, diabetes mellitus, and LVEF were not significantly different between the two groups, as were the intraoperative parameters of surgery type and UFV. However, the cardiac function in basic parameters, operation time, CPB time and clamp time in intraoperative parameters emerged huge increase in ALI patients by comparing with non-ALI counterparts. Further, the perioperative risk factors including MV time, ICU LOS, and hospital LOS were evidently prolonged in ALI patients.

**TABLE 1:** Demographic and clinical characteristics of the patients enrolled in the study cohort.

	ALL (*n* = 70)	ALI (*n* = 20)	Non-ALI (*n* = 50)	*P*-value
**Basic parameters**				
Male (*n*)	37 (52.9%)	11 (55.0%)	26 (52.0%)	0.820
Age (years)	69.00 (58.75, 72.25)	71.50 (68.00, 72.75)	66.00 (57.00, 72.50)	0.073
Body mass index	24.84 ± 3.84	25.18 ± 4.22	24.71 ± 3.72	0.646
Smoking (*n*)	28 (40.0%)	6 (30.0%)	22 (44.0%)	0.280
Hypertension (*n*)	49 (70.0%)	17 (85.0%)	32 (64.0%)	0.083
Diabetes mellitus (*n*)	30 (42.9%)	5 (25.0%)	25 (50.0%)	0.056
Cardiac function (NYHA)	3.00 (2.88, 3.00)	3.00 (3.00, 3.50)	3.00 (2.00, 3.00)	**0.004**
LVEF (%)	63.00 (59.00, 68.00)	62.00 (59.25, 64.00)	64.00 (59.00, 69.00)	0.159
**Intraoperative parameters**				
Surgery type	–	–	–	0.934
Coronary artery surgery (%)	37 (52.9%)	11 (55%)	26 (52%)	–
Valve surgery (%)	24 (34.3%)	6 (30%)	18 (36%)	–
Congenital heart surgery (%)	3 (4.3%)	1 (5%)	2 (4%)	–
Vascular surgery (%)	6 (8.6%)	2 (10%)	4 (8%)	–
Operation time (min)	327.43 ± 39.55	361.25 ± 23.22	313.90 ± 36.61	**<0.001**
CPB time (min)	138.67 ± 23.81	157.50 ± 20.44	131.14 ± 20.82	**<0.001**
Clamp time (min)	105.69 ± 22.36	117.55 ± 20.80	100.94 ± 21.35	**0.004**
UFV (ml)	3250.00 (2150.00, 4125.00)	3400.00 (1550.00, 4250.00)	3150.00 (2200.00, 4125.00)	0.735
**Peri-operative risk factors**				
MV time (h)	18.00 (15.22, 22.00)	40.50 (18.50, 46.88)	17.50 (11.50, 18.78)	**<0.001**
ICU LOS (days)	2.75 (1.81, 3.63)	3.69 (2.73, 3.93)	2.02 (1.79, 2.84)	**0.001**
Hospital LOS (days)	23.61 ± 6.47	27.48 ± 7.18	22.07 ± 5.52	**0.001**

Data are presented as number of patients (%), median (interquartile range), or counts, as appropriate. ALI, acute lung injury; CPB, cardiopulmonary bypass; UFV, ultrafiltered volume removal; LOS, length of stay; MV, mechanical ventilation; ICU, intensive care unit.

Results with statistical differences (*p* < 0.05) are bold.

### Perioperative serum calpains’ concentrations

**FIGURE 1:**
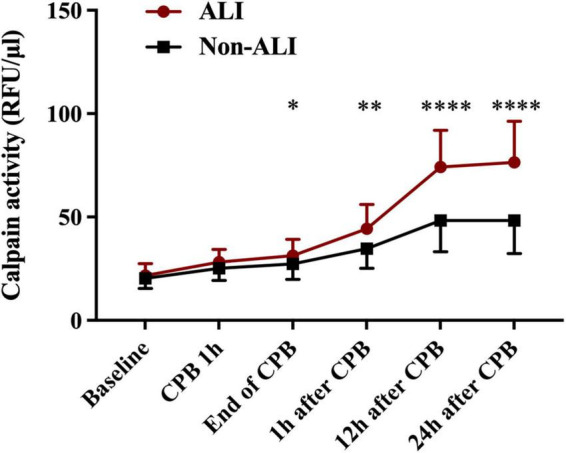
Mean serum concentration of serum level of calpains at baseline level and different time points perioperative period. (*p < 0.05, **p < 0.01, and ****p < 0.0001).

Postoperative serum calpains’ concentrations are shown in Figure 1 and Table 2. Difference in serum level of calpains between the two groups first appeared at the end of CPB, and at the following detection time points within 24 h, the serum level of calpains in ALI patient was obviously higher than that in non-ALI counterparts.

**TABLE 2:** Serum calpains’ activity relative fluorescence unit (RFU/μl) at baseline level and different time points after cardiopulmonary bypass (CPB).

Calpains’ activity (RFU/μ l)	ALI (*n* = 20)	Non-ALI (*n* = 50)	*P*-value
Beginning of CPB (baseline)	20.07 (16.69, 25.47)	19.19 (16.95, 23.36)	0.317
1 h during CPB	24.97 (23.82, 31.58)	24.26 (20.83, 29.71)	0.095
End of CPB	29.33 (24.37, 38.45)	25.80 (21.16, 31.21)	**0.048**
1 h after CPB	44.32 ± 11.76	34.64 ± 9.50	**0.001**
12 h after CPB	74.14 ± 17.80	48.29 ± 15.06	**<0.001**
24 h after CPB	78.03 (55.22, 90.60)	44.72 (36.00, 60.10)	**<0.001**

Data are presented as mean ± SD or median (interquartile range), as appropriate.

Results with statistical differences (*p* < 0.05) are bold.

### Serum level of calpains at 1 h after cardiopulmonary bypass as a predictor for postoperative acute lung injury

Univariate logistic regression analysis including cardiac function, operation time, CPB time, clamp time as well as serum level of calpains at the end of CPB and 1 h after CPB. It revealed that cardiac function, operation time, CPB time, clamp time, and serum level of calpains at 1 h after CPB were conspicuous related with an increased risk of ALI. After adjustment by multivariable logistic analysis, cardiac function, operation time, CPB time, and serum calpains at 1 h after CPB were highly associated with the incidence of postoperative ALI (Table 3).

**TABLE 3:** Univariate and multivariate analysis of serum level of calpains after cardiopulmonary bypass (CPB) for prediction of acute lung injury (ALI).

	Univariate		Multivariate	
		
	OR (95% CI)	*P*-value	OR (95% CI)	*P*-value
Cardiac function (NYHA)	6.146 (1.608, 23.484)	0.008	74.171 (2.067, 2660.883)	**0.018**
Operation time (min)	1.048 (1.024, 1.071)	<0.001	1.084 (1.024, 1.147)	**0.005**
CPB time (min)	1.071 (1.032, 1.112)	<0.001	1.088 (1.001, 1.181)	**0.046**
Clamp time (min)	1.040 (1.010, 1.070)	0.008	1.022 (0.943, 1.106)	0.599
End of CPB (RFU/μl)	1.003 (1.000, 1.007)	0.061	0.999 (0.988, 1.010)	0.835
1 h after CPB (RFU/μl)	1.004 (1.002, 1.007)	0.002	1.011 (1.001, 1.021)	**0.033**

Results with statistical differences (*p* < 0.05) are bold.

**FIGURE 2:**
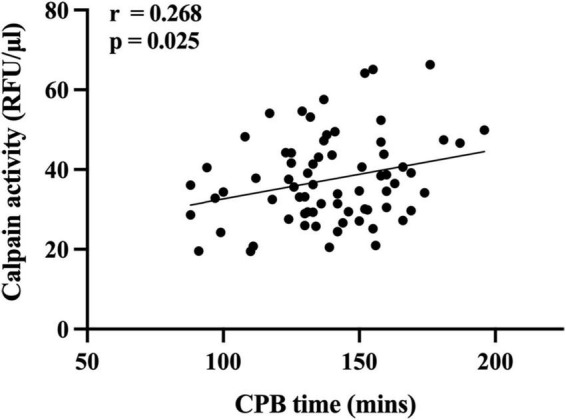
Scatterplot displaying the relation between cardiopulmonary bypass (CPB) time and serum level of calpains 1 h after CPB.

**FIGURE 3:**
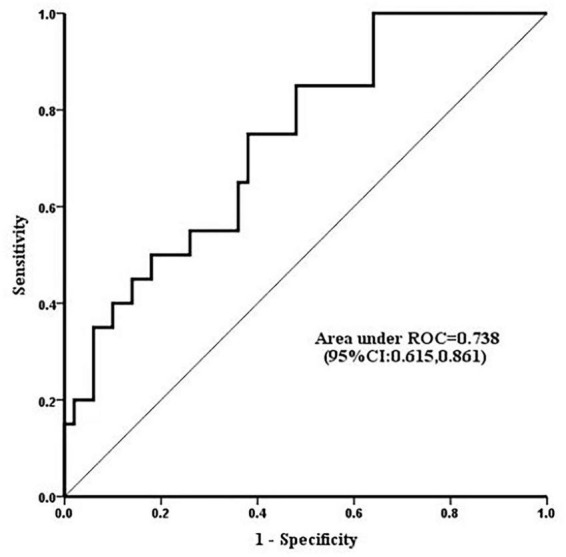
Receiver operating characteristic curves displaying the ability of serum level of calpains 1 h after cardiopulmonary bypass (CPB) to predict the occurrence of acute lung injury (ALI). The area under the curve (AUC) of serum calpains’ level 1 h after CPB for ALI was 0.738 (95% CI 0.615, 0.861).

Serum level of calpains at 1 h after CPB correlated with CPB time (Figure 2), and in ROC analysis, the AUC of serum level of calpains at 1 h after CPB for ALI was 0.738 (Figure 3). Derived sensitivity, specificity, and predictive cutoff value of serum level of calpains at 1 h after CPB was listed in Table 4. The serum level of calpains at 1 h after CPB had the highest sensitivity and specificity at the cutoff value of 33.52 (RFU/μl).

**TABLE 4:** Performance of serum level of calpains 1 h after cardiopulmonary bypass (CPB) (RFU/μl) for diagnosis of acute lung injury (ALI).

	Area under ROC curve (95% CI)	At cutoff value of 33.52 (RFU/μl)			
		
		Sensitivity (95% CI)	Specificity (95% CI)	PPV (95% CI)	NPV (95% CI)
1 h after CPB (RFU/μl)	0.738 (0.615, 0.861)	0.850 (0.694, 1.000)	0.520 (0.382, 0.658)	0.415 (0.264, 0.566)	0.897 (0.300, 1.000)

### Serum calpains at 1 h after cardiopulmonary bypass predict a severity of acute lung injury and poor clinical outcomes

**FIGURE 4:**
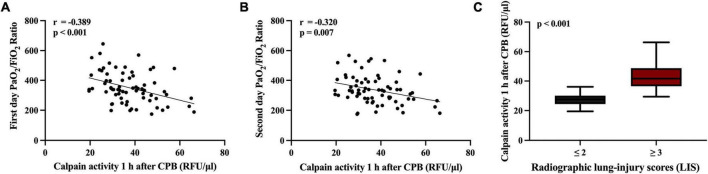
Scatterplot displaying the relation between serum level of calpains 1 h after cardiopulmonary bypass (CPB) and the inspiratory oxygen fraction to oxygen pressure (PaO_2_/FiO_2_) ratio at first 2 days **(A,B)**, and radiographic lung-injury scores (LIS) **(C)**.

**FIGURE 5:**
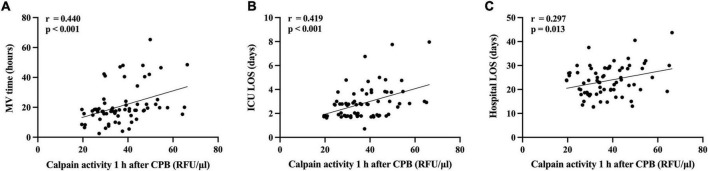
Scatterplot displaying the relation between serum level of calpains 1 h after cardiopulmonary bypass (CPB) and the clinical outcomes. The mechanical ventilation time **(A)**, the intensive care unit (ICU) length of stay (LOS) **(B)**, and the hospital LOS **(C)** are shown.

Higher serum calpains’ level at 1 h followed CPB were significantly related with more a severe ALI, as reflected by the lower PaO_2_/FiO_2_ ratio measured in the first two postoperative days (Figures 4A,B) and higher radiographic LIS (Figure 4C). Likewise, elevated serum calpains were correlated with poor clinical outcomes, as reflected by the longer MV time (Figure 5A), ICU LOS (Figure 5B), and hospital LOS (Figure 5C).

## Discussion

In current prospective pilot study of perioperative serum level of calpains in patients undergoing cardiac surgery using CPB technique, serum calpains’ concentrations were remarkably elevated after CPB and were paralleled with the development of ALI as well as clinical outcomes. According to the dynamic measurements, serum calpains’ level in the ALI patients present a prominent increase by comparing with non-ALI counterparts within 24 h after CPB, indicating that serum calpains’ level may serve as a potential biomarker for postoperative ALI.

Dynamic detection of serum calpains was started from the beginning of CPB. With the progress of CPB, the serum calpains’ level increased in all patients. The significant difference between two groups appeared at the end of CPB. Further, the difference has been maintained based on detection results at test time points up to 24 h after CPB. We selected two indicators of the earliest difference time points (serum level of calpains at the end of CPB and 1 h after CPB) for further logistic regression analysis, and the results suggested that only the serum level of calpains at 1 h after CPB has predictive value. Thus, the serum level of calpains at 1 h after CPB could serve as the first predictor of postoperative ALI. In the following analysis, serum calpains were related with the impaired lung function such as lower PaO_2_/FiO_2_ and LIS as well as poor clinical outcomes such as longer MV time, ICU LOS, and hospital LOS.

Mechanisms of CPB related lung injury include inflammation and ischemia-reperfusion injury. Both pathological processes can lead to the upregulation of intracellular Ca^2+^ (15, 16), which implies the activation of calpains. Calpains can also give positive feedback to the above two pathological processes. On one hand, calpains play an important role in inflammation. By participating in the activation of NF-κB signaling pathway, calpain could promote the expression of pro-inflammatory cytokines and adhesion molecules (23, 24). In addition, cleavage of junctional proteins by calpains promote the infiltration of inflammatory cells (18–25). Finally, calpains can be externalized during inflammation and function in the microenvironment (18). On the other hand, the ischemic process during CPB leads to mechanical stress loss of pulmonary vessels, resulting in a large influx of Ca^2+^ (13). In the subsequent reperfusion phase, oxygenated blood re-influxes into the lung tissue, leading to the massive production and release of oxygen free radicals and more severe calcium overload (26), processes that activate calpains and amplifies cellular damage (27, 28). The increase of calpains has been proved and to exert a negative effect during ischemia-reperfusion (28–31). In an animal model of CPB, inhibition of calpains is beneficial, which reduces endothelin-1 expression and maintains eNOS levels and, therefore, results in lower pulmonary vascular resistance (32). The role of calpains in endothelial barrier is highlighted (25, 33), up-regulation of calpains could proteolysis the juxta-membrane domain of VE-cadherin, thereby destabilizing the adhesive junction and promoting the infiltration of inflammatory cells (33), which is an important hallmark of ALI (34, 35). Notably, calpains were also found in lung microvascular cells to hydrolyze VE-cadherin, disrupting the endothelial barrier, and promoting pulmonary edema (36).

Calpains are normally perform regulatory functions intracellularly. The discovery of calpains externalization further refines their biological functions in organisms (17). Recent studies suggest that calpains are externalized through two distinct mechanisms: autonomous secretion and leakage from dying cells. Letavernier et al. noted that externalization of calpains’ activity increased when endothelial cells were exposed to VEGF-A and norepinephrine (37). Secretory modalities include cellular vesicles (38) or, *via* the ATP-binding cassette transporter A1 (ABCA1) (39). In the other way of externalization, calpains released from dying cells. The mechanism of externalized calpain is ambiguous. Externalized calpain could promote angiogenesis by cleaving fibronectin, accelerating the organ repair (37). However, in Limaye et al. study on acute liver injury, externalized calpain was found mediating damage to surrounding normal hepatocytes through hydrolyzing cytoskeleton proteins (40). The ubiquitously expressed of calpains make it difficult to trace the origin. In the present research, we monitored the parameters of other organs such as liver and kidney to evaluate whether high level of serum calpains could also reflect liver and kidney injury after CPB. However, the data showed that hyperactive calpains did not cause similar damage to other organs except lungs (Supplementary Table 1), which partly confirmed the specificity of serum calpains on lung injury.

The limitations of this study should be acknowledged. First, based on a single-center study, the insufficient number of enrolled patients may limit the application of our relatively correlated findings in other institutions. Hence, larger cohort studies to further validate these results were needed. Second, the detection of inflammatory cytokines was not quantified in this trial, which limited our research on the correlation between calpains and inflammatory cytokines. Third, it is difficult to determine whether elevated serum level of calpains is a cause of ALI or a consequence of CPB which leading to ALI. In this case, ALI people who have not experienced CPB need to be included in the comparison.

## Conclusion

In current clinical trial, the potential role of serum calpains in predicting the incidence of postoperative ALI followed CPB was identified. The predictive value of which was shown as early as 1 h after CPB. At the same time, serum calpains were associated with poorer clinical outcomes. Afterall, an in-depth understanding of the role of calpains in clinical manifestations and development has the potential to better understanding of CPB-induced ALI.

## Data availability statement

The original contributions presented in this study are included in the article/Supplementary material, further inquiries can be directed to the corresponding authors.

## Ethics statement

The studies involving human participants were reviewed and approved by Medical Ethical Committee of Affiliated Hospital of Yangzhou University. The patients/participants provided their written informed consent to participate in this study. Written informed consent was obtained from the individual(s) for the publication of any potentially identifiable images or data included in this article.

## Author contributions

WW, QW, and XW formulated the idea of the manuscript and supervised the research. CY, FC, and RM performed the research and wrote the manuscript. CY, ZF, WW, and QW carried out the operations. RM, LC, and XT collected the clinical samples. MS, QZ, QS, and GZ participated in preparing the figures, tables, and data analyzing. PL and BL provided comments and technical advice. WW and XW revised the manuscript and provided comments. All authors reviewed the manuscript.
