# Supplementary material for: Serum level of calpains product as a novel biomarker of acute lung injury following cardiopulmonary bypass
Source: Front Cardiovasc Med. 2022 Nov 16;9:1000761. doi: 10.3389/fcvm.2022.1000761 (PMC9709320; doi:10.3389/fcvm.2022.1000761)
Supplement: Supplementary file 1 [file Data_Sheet_1.pdf]

Supplementary Material

Supplementary Figure. CONSORT patient flow diagram.

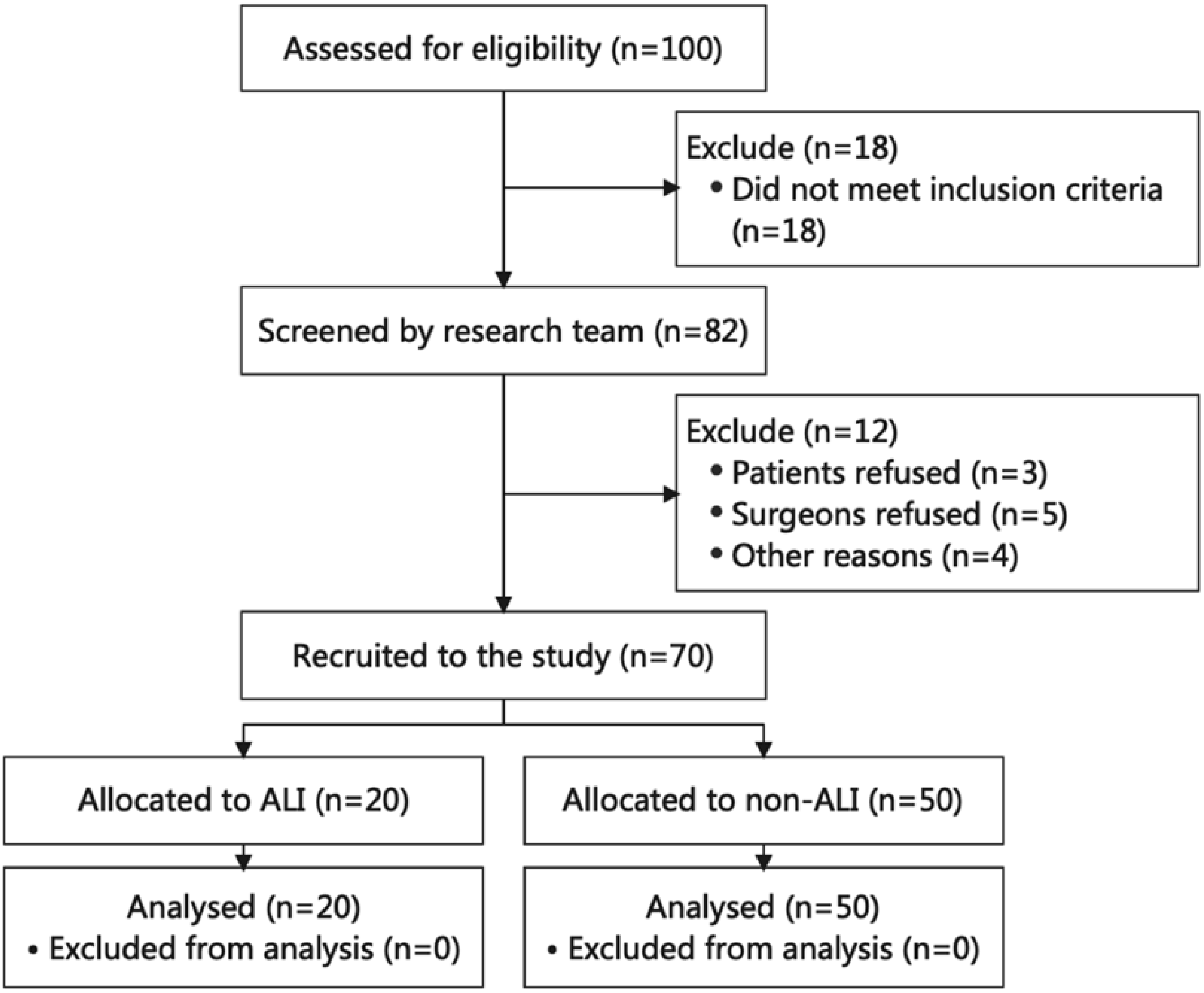

Supplementary Table 1：Liver and kidney function of the patients enrolled in the study cohort

	ALI (n = 20)	Non-ALI (n = 50)	P value
**ALT (U/L)**			
1 h after CPB	32.1 ± 14.45	29.00 (23.75, 46.50)	0.369
12 h after CPB	29.00 (25.00, 49.75)	29.50 (23.75, 46.50)	0.888
24 h after CPB	31.50 (20.5, 49.75)	27.00 (21.00, 41.00)	0.466
**AST (U/L)**			
1 h after CPB	53.80 ± 21.27	45.50 (38.00, 58.75)	0.589
12 h after CPB	72.50 (38.75, 84.00)	46.50 (38.00, 57.50)	0.101
24 h after CPB	54.50 (29.00, 86.25)	35.00 (26.75, 54.25)	0.084
**BUN (mg/dl)**			
1 h after CPB	6.92 ± 2.66	5.07 (4.27, 7.20)	0.138
12 h after CPB	6.29 (5.32, 9.64)	6.28 (5.17, 8.25)	0.511
24 h after CPB	9.93 ± 3.98	6.86 (5.73, 10.39)	0.125
**Scr (μmol/L)**			
1 h after CPB	82.10 (63.93, 100.18)	66.45 (57.48, 88.48)	0.128
12 h after CPB	102.35 (80.88, 132.30)	87.60 (69.60, 106.05)	0.077
24 h after CPB	104.10 (75.95, 134.13)	84.45 (61.95, 107.28)	0.054

Data are presented as number of patients (%), median (interquartile range), or counts, as appropriate. ALT, Alanine aminotransferase; AST, Aspartate aminotransferase; BUN, blood urea nitrogen; Scr, serum creatinine.
